# Pattern of sucker development in cuttlefishes

**DOI:** 10.1186/s12983-020-00371-z

**Published:** 2020-08-24

**Authors:** Ryosuke Kimbara, Mayuko Nakamura, Kohei Oguchi, Hisanori Kohtsuka, Toru Miura

**Affiliations:** 1 Misaki Marine Biological Station, School of Science, The University of Tokyo, Miura, Kanagawa 238-0225 Japan; 2 The National Institute of Advanced Industrial Science and Technology (AIST), Tsukuba, Ibaraki, 305-8566 Japan

**Keywords:** Cephalopod, Cuttlefish, Novelty, Sucker, Embryogenesis, Postembryonic development, Sucker buds, Sucker field ridge

## Abstract

**Background:**

Morphological novelties have been acquired through evolutionary processes and related to the adaptation of new life-history strategies with new functions of the bodyparts. Cephalopod molluscs such as octopuses, squids and cuttlefishes possess unique morphological characteristics. Among those novel morphologies, in particular, suckers arranged along the oral side of each arm possess multiple functions, such as capturing prey and locomotion, so that the sucker morphology is diversified among species, depending on their ecological niche. However, the detailed developmental process of sucker formation has remained unclear, although it is known that new suckers are formed or added during both embryonic and postembryonic development. In the present study, therefore, focusing on two cuttlefish species, *Sepia esculenta* and *S. lycidas*, in which the sucker morphology is relatively simple, morphological and histological observations were carried out during embryonic and postembryonic development to elucidate the developmental process of sucker formation and to compare them among other cephalopod species.

**Results:**

The observations in both species clearly showed that the newly formed suckers were added on the oral side of the most distal tip of each arm during embryonic and postembryonic development. On the oral side of the arm tip, the epithelial tissue became swollen to form a ridge along the proximal-distal axis (sucker field ridge). Next to the sucker field ridge, there were small dome-shaped bulges that are presumed to be the sucker buds. Toward the proximal direction, the buds became functional suckers, in which the inner tissues differentiated to form the complex sucker structures. During postembryonic development, on both sides of the sucker field ridge, epithelial tissues extended to form a sheath, covering the ridge for protection of undifferentiated suckers.

**Conclusions:**

The developmental process of sucker formation, in which sucker buds are generated from a ridge structure (sucker field ridge) on the oral side at the distal-most arm tip, was shared in both cuttlefish species, although some minor heterochronic shifts of the developmental events were detected between the two species.

(325 words)

## Background

**Fig. 1:**
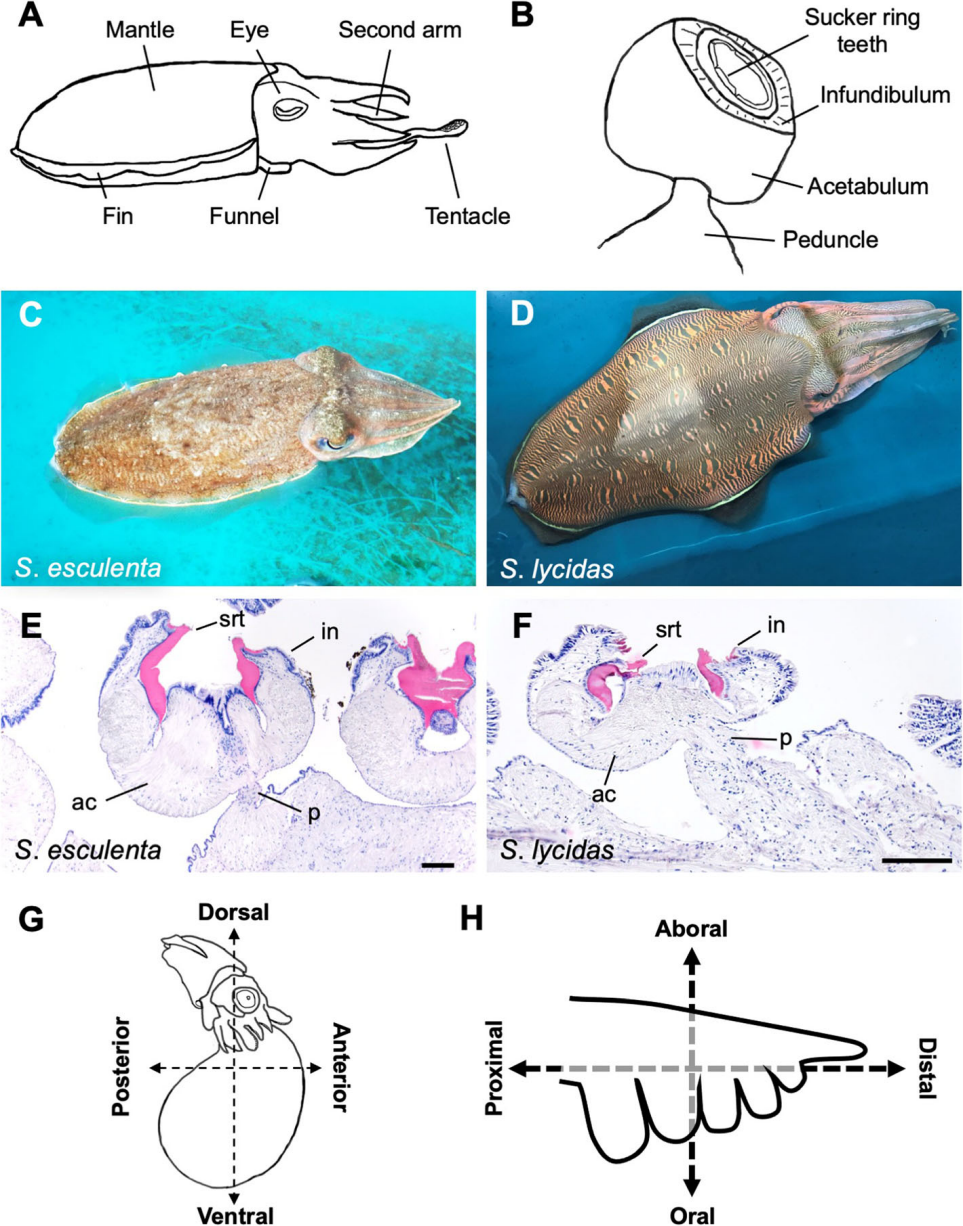
The general morphology, embryonic development, and the definition of axes in cuttlefishes. **a** A schematic image of the cuttlefish body plan. **b** The general structure of the sucker of Decapodiformes. **c** The golden cuttlefish *Sepia esculenta.* **d** The kisslip cuttlefish *S. lycidas*. **e**, **f** Histological sections of the sucker in adult cuttlefishes. **e** *S. esculenta*. **f** *S. lycidas*. **g** The definition of the body axis of an embryo used in this study. **h** The definition of the axis of an arm. Scale bars: 200 μm. Abbreviations: ac, acetabulum; in, infundibulum; p, peduncle; srt, sucker ring teeth

In evolution, the acquisition of novel characteristics is known to play crucial roles in adaptation to new environments [1]. Among bilaterians, the bodyplans of lophotrochozoans are the most diversified [2]. Especially, molluscs exhibit diverse and unique characteristics, including their bodyplans [3, 4], and cephalopods (class Cephalopoda) are the most distinctive group in molluscs [3, 5]. They show highly developed brains [6], ink sacs [7], and chromatophores [8]. Extant cephalopod molluscs (class Cephalopoda) are composed of 2 subclasses, Nautiloidea and Coleoidea, and Coleoidea is classified into 2 superorders, i.e., Decapodiformes (squids and cuttlefishes) and Octopodiformes [5, 9]. Numerous arms and suckers on arms are also important novelties in cephalopods (Fig. 1a), although the basal cephalopod lineages, i.e., Nautiloidea, lack suckers [10]. Since cephalopod arms are used not only for locomotion but also for prey capture [11], copulation [12], and oviposition [13], the suckers may have adapted to contribute to these arm functions [14].

Especially in Decapodiformes, the sucker morphology is diversified among species [15]. Basically, an adult sucker is composed of an attachment face (infundibulum), a chamber for producing suction (acetabulum), and a peduncle by which the acetabulum is attached to the arm surface, and there is a proteinaceous ring with teeth on the rim of the infundibulum (sucker ring teeth) (Fig. 1b) [16, 17]. In some species, furthermore, suckers are modified into hooks, losing the sucking functions [18]. Thus, the sucker morphology is deeply related to life-history strategies and the adaptive radiation in cephalopods [14, 18, 19].

There have so far been several studies on the development of arms in Decapodiformes [20, 21]. It was reported that, in Decapodiformes, the number of suckers increases as arms are elongated during postembryonic development [10]. However, there are few studies focusing on the developmental process of sucker formation throughout these animals’ lifetime. In the embryonic development of *Euprymna scolopes* (Sepiolida), small undifferentiated suckers were observed on the oral side of the arm tip, although the process of sucker development was not described in detail [20]. In some squid species belonging to Myopsida and Oegopsida, the processes of embryonic development were described (*Illex illecebrosus*: [22]; *Sepioteuthis lessoniana*: [23]; *Dosidicus gigas*: [24]; *Todarodes pacificus*: [25]), but the descriptions did not focus on sucker formation. Thus, the pattern of sucker formation during embryonic and postembryonic development has so far been poorly understood.

In this study, therefore, to understand the developmental process of cephalopod suckers, the pattern of sucker formation during development was investigated, focusing on 2 cuttlefish species belonging to the order Sepiida, i.e., *Sepia esculenta* and *S. lycidas* (Fig. 1c, d). In Sepiida, which is thought to be an early-branched group among Decapodiformes (although there are several hypotheses about this [26, 27]), the sucker morphology is relatively simple without specialized structures such as hooks [15] and is thought to show an ancestral state with some species diversity. Between the two focal cuttlefish species, few or no differences were observed in the sucker structures (Fig. 1e, f), although the size difference was seen. Sepiida cuttlefishes are relatively easy to rear after hatching because they are benthic, while other groups of squids are pelagic and difficult to maintain in the laboratory [28]. The 2 focal Sepiida species are easily available and they lay many large eggs that are suitable for embryonic observations. The reproductive season of the two species is overlapped but a little bit different, so that developing embryos can be obtained from the two species for a longer period. Furthermore, we expected that the comparison between the two species would tell us about the shared mechanisms underlying the sucker development.

In the focal species, the embryonic stages can be identified based on morphological features, according to a previous study in *Sepiella japonica* [29] (Fig. S1, Table 1). In this study, the body axes of embryos and arms were defined according to the previous study (Fig. 1g, h) [20]. For postembryonic development, mantle length (ML) was utilized as a criterion of growth because the growth speed could be variable among individuals, so the time period elapsed during development is not suitable as a growth index [30, 31]. Among the four pairs of arms and the pair of tentacles, the second arms were focused on in this study, since they are the simplest arms, while the others exhibit specialized morphologies like swimming arms or tentacles.

**Table 1:** Developmental stages of the cuttlefish. Main developmental events at each stage are based on [29]

Stage	Main developmental events
24–25	Head with eye vesicles protrude laterally. Fins are evident on the mantle. Mantle is beginning to grow downward. Shell is discernible with some difficulty. Fourth arm (tentacle) elongates prominently.
26–27	Eye vesicles are nearly spherical. Lens primordia may be seen with care. Tentacle curves upward to backward. Funnel folds begin to form the siphon. Sucker primordia are present on the arms.
28–29	Pigmentation has begun in the retina (very pale yellow to light orange). Lens primordium is prominent as a refractive rod. Iris fold is forming pupil. Median margins of the funnel folds are fusing. Tentacle is coiled once. Heart beat may be discernible with some difficulty. Organ of Hoyle (T-shaped thickening on the dorsal surface of the mantle) is visible faintly. Heart beat is evident.
30–32	Retina is orange to reddish brown. Pigmentation of iris has occurred. Reflective dots appear in the shell. Tentacle coiled twice. Chromatophores present in the mantle. Organ of Hoyle evident.
33–36	Retina is brownish red to reddish black. Eye ball gradually becomes iridescent and opaque. Two to four striations appear on the dorsal surface of the shell. Pigmentation of ink sac occurs. Secondary cornea covers the eye. Tentacle is uncoiled and peduncle of tentacle is retracted into the tentacular sac by degrees.
37–38	Eye is black and strongly iridescent. Lid occurs at the margin of the pupil and covers the pupil. Five to six striations are on the shell. Iridescent layer is evident on the ink sac. Whole tentacle is retracted into the tentacular sac. Slight pigmentation is discernible in the jaws. Iridescence is evident on the surface of the mantle.
39	Seven striations are on the shell. Yolk sac prominently decreases in size.

Since it was predicted that functional suckers with differentiated structures were developed from undifferentiated buds, observations on the external morphology and internal histological structures of cuttlefish arms during embryonic and postembryonic development were carried out to elucidate the pattern of sucker formation. Generally, in Coleoidea (octopuses, squids and cuttlefishes), small suckers are located at the distal tips of arms, indicating that the new suckers are formed at the distal tips, as suggested in a few species [20, 21]. In this study, therefore, the distal arm tips were focused on.

## Results

### Increase of suckers throughout development

**Fig. 2:**
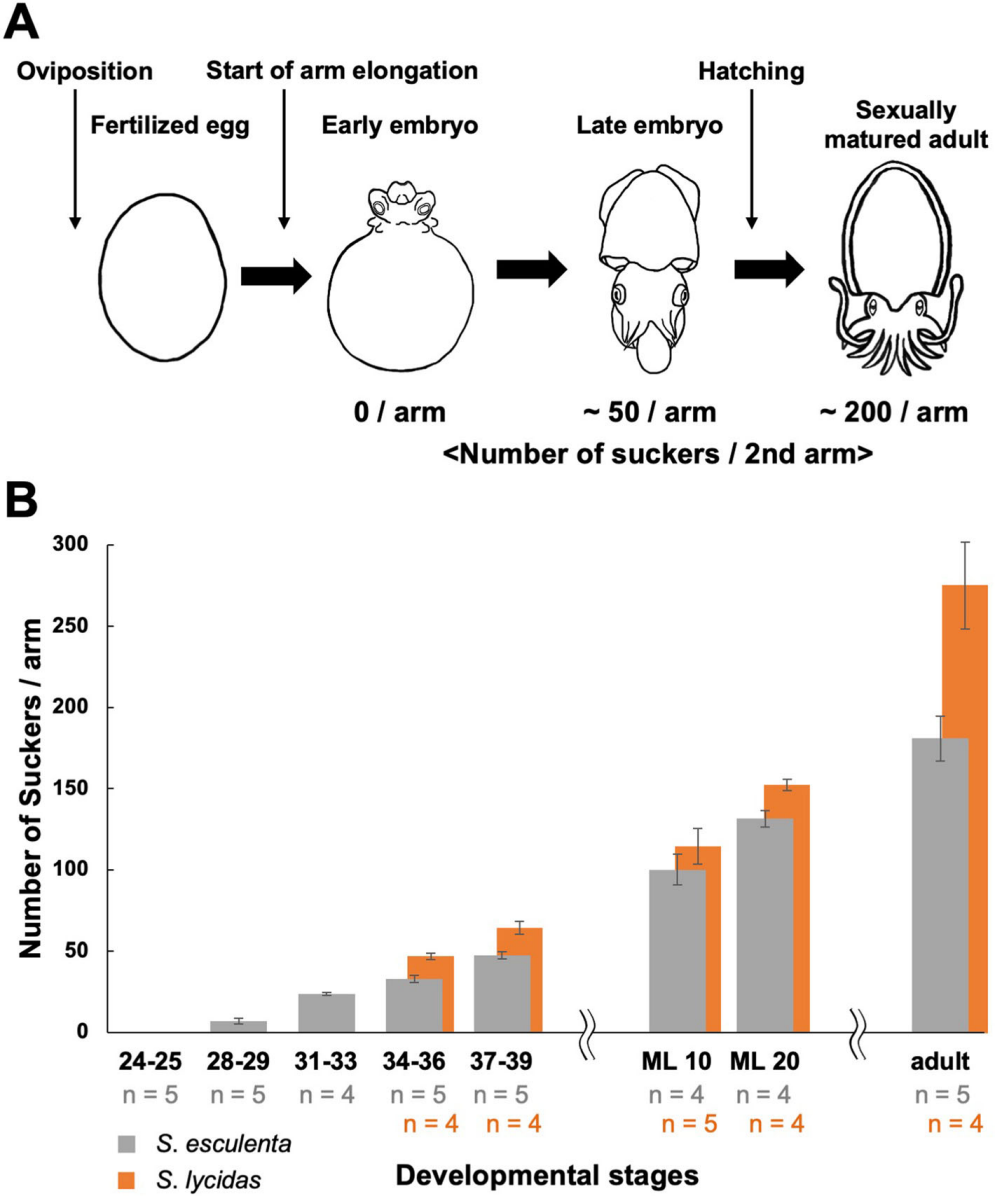
The number increase of suckers on the second arm during the growth in cuttlefishes. **a** Schematic images of cuttlefish development, together with the corresponding sucker number in *S. esculenta*. **b** The transition of sucker number on the second arm of *S. esculenta* and *S. lycidas* during embryonic and postembryonic development. Gray bars: *S. esculenta*; orange bars: *S. lycidas*. Number under each column indicates the sample size (gray numbers: *S. esculenta*; orange numbers: *S. lycidas*). Error bars indicate standard deviation

Firstly, to elucidate the increase pattern of suckers in *S. esculenta* and *S. lycidas*, the number of suckers on the second arm were counted in embryos, juveniles and adults. No suckers were observed on arms in embryos at early developmental stages (St. 24–25), during which arms start to elongate, but they seemed to form at later stages. Moreover, in sexually matured adults, there were many more suckers than in embryos (Fig. 2a).

The number of suckers (including primordial suckers) on the second arm was counted in *S. esculenta*. The sucker number (including primordial suckers) per second arm (mean ± S.D.) was 7 ± 1.6 at St. 28–29, 24 ± 0.96 at St. 31–33, 33 ± 2.0 at St. 34–36, 47 ± 2.19 at St. 37–39, 100 ± 9.4 in ML10 juveniles (mantle length: 10 mm), 132 ± 5.1 in ML20 juveniles, and 181 ± 13 in sexually mature adults (Fig. 2b). Similar investigations were also carried out in *S. lycidas*, showing that the sucker number on the second arm was 45 ± 1.9 at St. 34–36, 62 ± 3.8 at St. 37–39, 110 ± 11 in ML10 juveniles (mantle length: 10 mm), 146 ± 3.4 in ML20 juveniles, and 263 ± 25 in sexually mature adults (mean ± S.D.). Interestingly, *S. lycidas* possessed more suckers than *S. esculenta*.

### Sucker formation during embryogenesis in *S. esculenta*

**Fig. 3:**
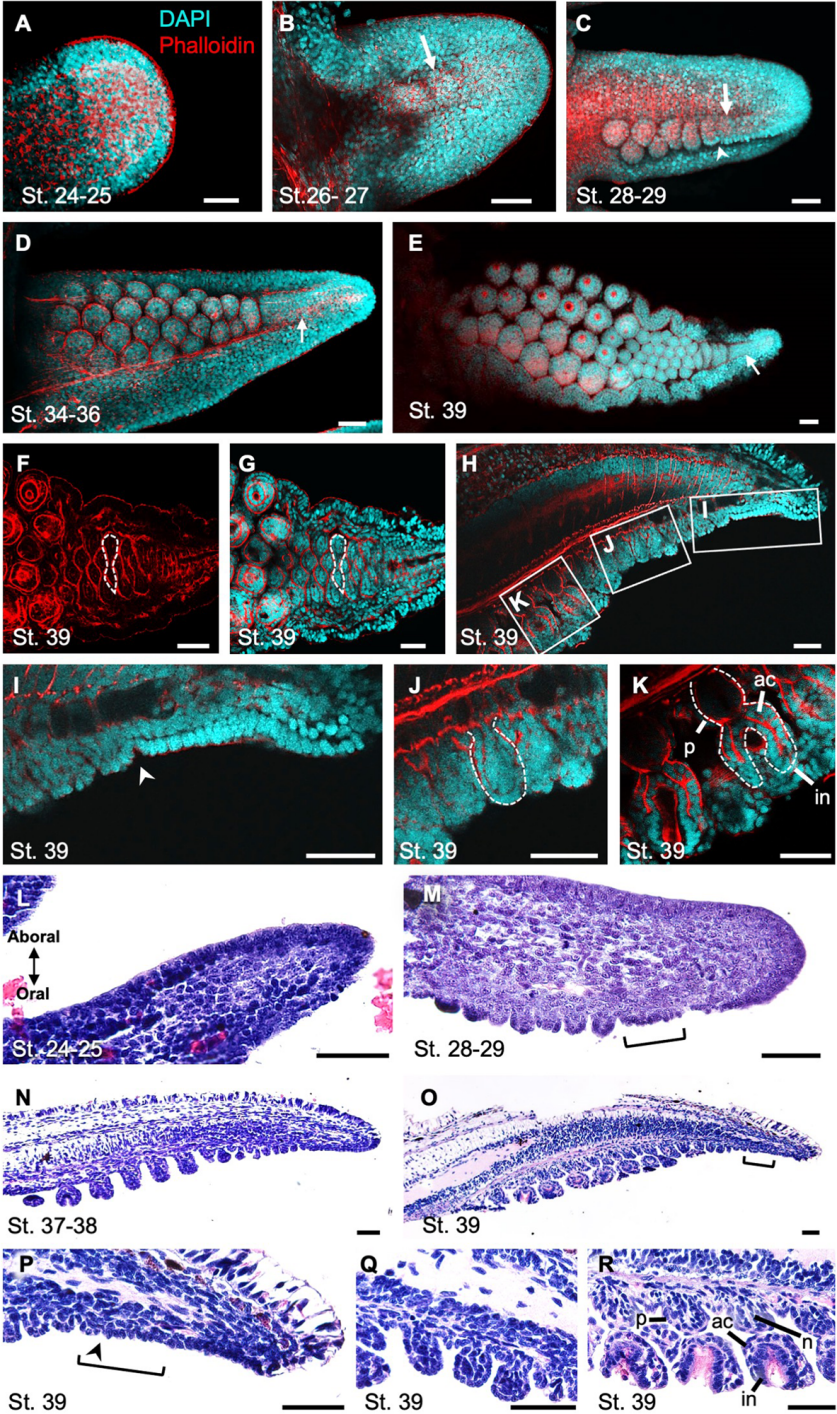
The process of sucker formation on the second arm of *S. esculenta* during embryonic development. Arms are oriented with distal to the right. **a**-**e** Confocal stacks of arms from oral view. Cyan: DAPI; red: phalloidin. Arrows: a long narrow ridge (sucker field ridge) on the arm tip. Arrowheads: the proximal constriction of the sucker field ridge. **a** St. 24–25 (*n* = 4). **b** St. 26–27 (*n* = 5). **c** St. 28–29 (n = 4). **d** St. 34–36 (n = 5). **e** St. 39 (n = 5). **f**, **g** Optical sections of the base of the buds at St. 39, stained with phalloidin (**f**), or DAPI/phalloidin (**g**) (n = 5). White dotted frames indicate the gourd-shaped actin localization. **h** A sagittal optical section at St. 39 (n = 5). **i**-**k** Higher magnification of white boxed regions in **h**. White dotted frames indicate the constricted sucker buds (**j**) and the primordial sucker with differentiated structures (**k**). **l**-**r** Histological sections in sagittal planes stained with hematoxylin and eosin. Arms are oriented with oral to the bottom. **l** St. 24–25. **j** St. 28–29. **n** St. 37–38. **o** St. 39. **p**-**r** Higher magnification of the primordial suckers at St. 39; distal (**p**), middle (**q**) and proximal (**r**) parts. Brackets indicate the region where epithelial cells composed one layer. Scale bars: 50 μm. Abbreviations: ac, acetabulum; in, infundibulum; n: nerve; p, peduncle

To investigate the pattern of sucker formation based on observations on the external morphologies and internal structures, the nucleus and cytoskeleton (F-actin) were stained and observed using a confocal laser scanning microscope (CLSM). The observation results showed that, in St-25 embryos at the early stage of arm elongation, there were no structures of primordial suckers (Fig. 3a). At St. 26–27, epithelial tissues on the oral side of arm tips were observed to be swollen (Fig. 3b). At St. 28–29, on the oral side of the arm tip, the epithelial tissue formed a ridge along the proximal-distal axis (sucker field ridge). On the more proximal area of the oral side, next to the sucker field ridge, multiple dome-shaped bulges (sucker buds) were located aligned in one or two rows in the proximal-distal direction, and the number of such bulges was increased in the more proximal part of the arm (Fig. 3c). At the middle to late embryonic stages (after St. 32), it was found that the sucker field ridge was located at the tip of the oral side of the arm, and many small sucker buds were aligned on the proximal side of the sucker field ridge (Fig. 3d, e).

From the distal to the proximal part of the arm, the number of suckers per row increased up to four. To examine the pattern of sucker-row formation in detail, observations of optical sections obtained from the basal part of each sucker buds were carried out and revealed that two buds were pinched off from one another, forming a gourd shape (Fig. 3f, g; for other developmental stages see Additional file 2: Fig. S2). Two pairs of gourd-shaped units were arranged alternately, forming 4 rows of suckers. Each unit of sucker buds in the 4 rows was clearly shown by the F-actin localization revealed by staining with phalloidin, and consisted of 2 cell components: a single layer of epithelial cells and the inside cells, which possessed relatively large nuclei (Additional file 3: Fig. S3).

At the stage just before hatching (St. 39), the gradual succession of stages of the process of sucker formation was observed along the distal-proximal axis of the arm; immature sucker buds were at the more distal part while larger and well-developed suckers were at the more proximal part (Fig. 3h). While dome-shaped sucker buds were observed at the distal tip, primordial suckers at more proximal positions showed constricted shapes, but without any detailed structures (Fig. 3h-j). At the most proximal part of an arm, furthermore, the structure of primordial suckers resembled the functional suckers in adults, with an attachment surface, a cup-shaped structure, and a stalk connecting to the arm, that corresponded, respectively, to an infundibulum, an acetabulum and a peduncle (Fig. 3k). In the cup-shaped structure, a ring-shaped accumulation of F-actin was observed along the cup edge (Fig. 3e).

The inner structures of arm tips were observed histologically, utilizing paraffin sections. At St. 24–25, neither distal sucker field ridge nor dome-shaped sucker buds were observed on the arms. However, the arrangement of epithelial cells was different between the oral and aboral sides (Fig. 3l). On the aboral side, the epithelial cells were arranged in orderly alignment, while the arrangement of epithelial cells on the oral side appeared random and disordered (Fig. 3l). At St. 28–29, the epithelium at the tip of the oral side was single-layered and cells under the epithelium looked randomly arranged without any clear structures (Fig. 3m). The size of sucker buds gradually increased from distal to proximal parts. At St. 37–38, although the distal part with undifferentiated sucker buds was similar to those at the earlier stages (Fig. 3n), in the proximal part of the arm, the base of a primordial suckers was constricted, forming a cup-shaped structure, which was not observed at earlier stages. At St. 39, the distal arm tip was similar to that in the earlier stages; a sucker field ridge with a single epithelial layer, from which sucker buds were differentiated, and multiple sucker buds were observed (Fig. 3o-q). In the proximal part of the arm, the attachment surface and the cup-shaped structure (i.e., infundibulum and acetabulum) became more obvious, and nerve-like tissues were observed in the stalk of the cup structure, i.e., the peduncle (Fig. 3o, r).

### Sucker formation during embryogenesis in *S. lycidas*

**Fig. 4:**
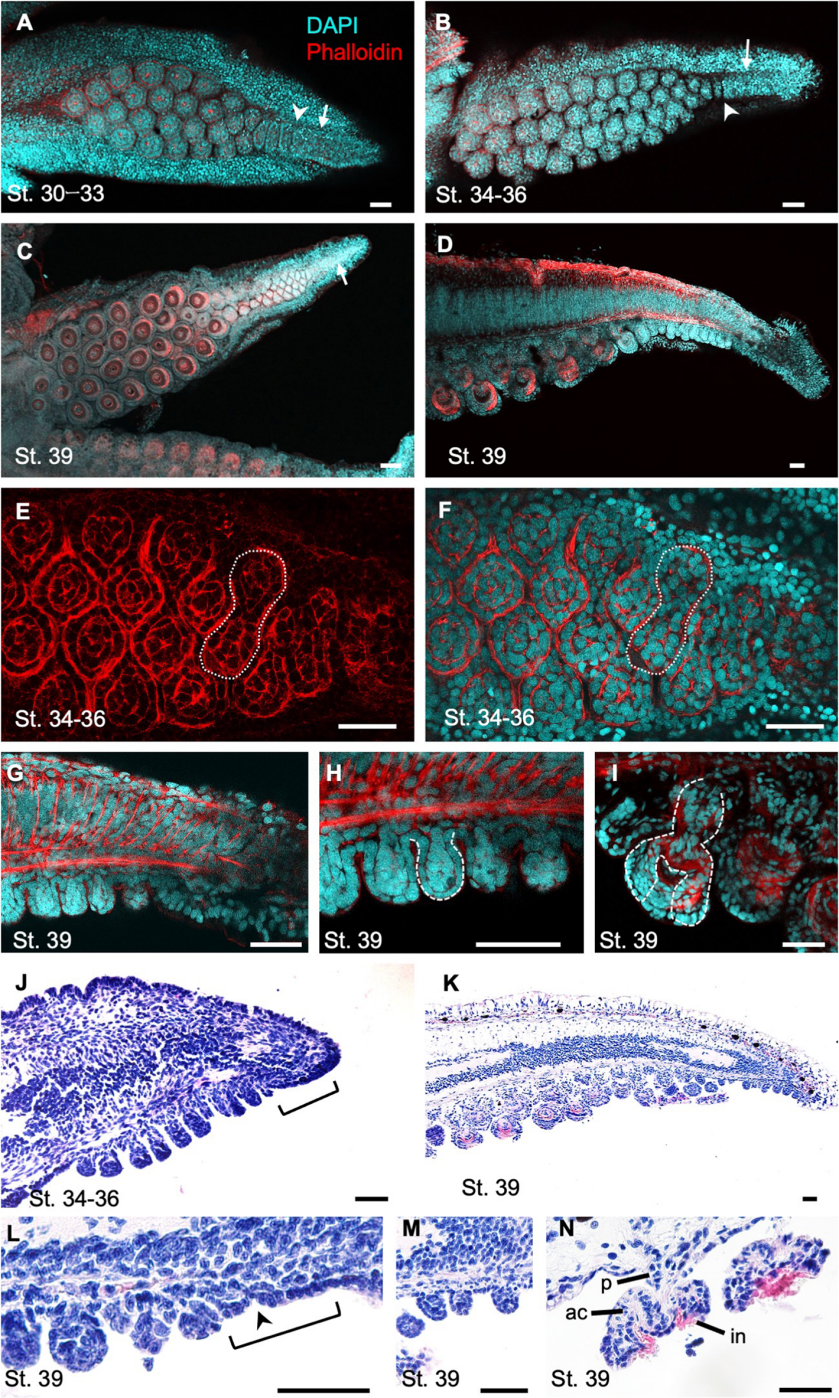
The process of sucker formation of the second arm in *S. lycidas* during embryonic development. Arms are oriented with distal to the right. **a**-**c** Confocal stacks of arms from oral view. Arrows indicate sucker field ridges on the arm tips. Arrowheads indicate the proximal constriction of the sucker field ridges. **a** St. 30–33 (*n* = 3). **b** St. 34–36 (n = 4). **c** St. 39 (n = 5). **d** A sagittal optical section at St. 39 (n = 5). **e**, **f** Optical sections of the base of sucker buds at St. 34–36 (n = 4). White dotted frames indicate the gourd-shaped actin localization. **g**-**i** the magnification of sagittal section; distal (**g**), middle (**h**), and proximal (**i**) parts. White dotted frames indicate the constricted sucker bud (**h**) and the primordial sucker with differentiated structures (**i**). **j**-**n** Histological sections in sagittal planes, stained with hematoxylin and eosin at St. 34–36 (**j**) and St. 39 (**k**). **l**-**n** Magnified images focusing on the primordial area at St. 39; distal (**l**), middle (**m**), and proximal (**n**) parts. Brackets indicate the regions where epithelial tissues are composed of one cell layer. Arrowheads indicate proximal constriction of sucker field ridges. Scale bars: 50 μm. Abbreviations: ac, acetabulum; in, infundibulum; p, peduncle

The sucker formation in *S. lycidas* was also observed to investigate whether the process seen in *S. esculenta* was shared among species. These observations showed that also in *S. lycidas*, a sucker field ridge was formed on the oral side of the arm tip along the proximal-distal axis, and a cluster of multiple dome-shaped sucker buds was observed in the more proximal part, adjacent to the sucker field ridge (Fig. 4a-d; St. 30–39). The number of primordial rows was only one at the distal arm tip, while it increased to two to four at more proximal positions. CLSM observations revealed that, as seen in *S. esculenta,* 2 buds were present in one unit with a gourd shape, and 4-sucker rows were formed by zig-zag alignment of these gourd-shaped units (Fig. 4e, f; for other developmental stages see Additional file 2: Fig. S2). At St. 39 (Fig. 4c, d), the size of primordial suckers was much larger in the proximal part than in the distal part of the arm, and an attachment surface (infundibulum), a cup-shaped structure (acetabulum) and a primordial stalk (peduncle) were clearly differentiated in the proximal part, whereas dome-shaped undifferentiated sucker buds were still observed in the distal part (Fig. 4g-i).

Histological observations in *S. lycidas* also showed that, at the arm tip, the most distal sucker field ridge with a single epithelial layer was observed on the oral side, from which dome-shaped sucker buds were differentiated from the proximal end (Fig. 4j, k; St. 34–39). At St. 39, in the distal part, dome-shaped sucker buds, in which the inner cell mass was undifferentiated, were similar to those seen at the earlier stages (Fig. 4l, m). In the proximal parts, each primordial sucker consisted of a cup-shaped structure and a stalk, which were presumed to develop into the adult sucker components (infundibulum, acetabulum and peduncle) (Fig. 4n).

Overall, in *S. lycidas,* both the morphology and the developmental pattern of suckers seemed similar to those in *S. esculenta*. However, focusing on the late embryonic stages (St. 34–39), the detailed sucker structures looked well-differentiated in *S. lycidas*, in comparison with those in *S. esculenta* (Figs. 3d, e, 4b, c*)*. Moreover, the sucker numbers rapidly increased from embryonic stages and the total number of suckers at adult stage was also larger in *S. lycidas* (Figs. 2b, 3a-e, 4a-c).

### Sucker formation during postembryonic development in *S. esculenta* and *S. lycidas*

**Fig. 5:**
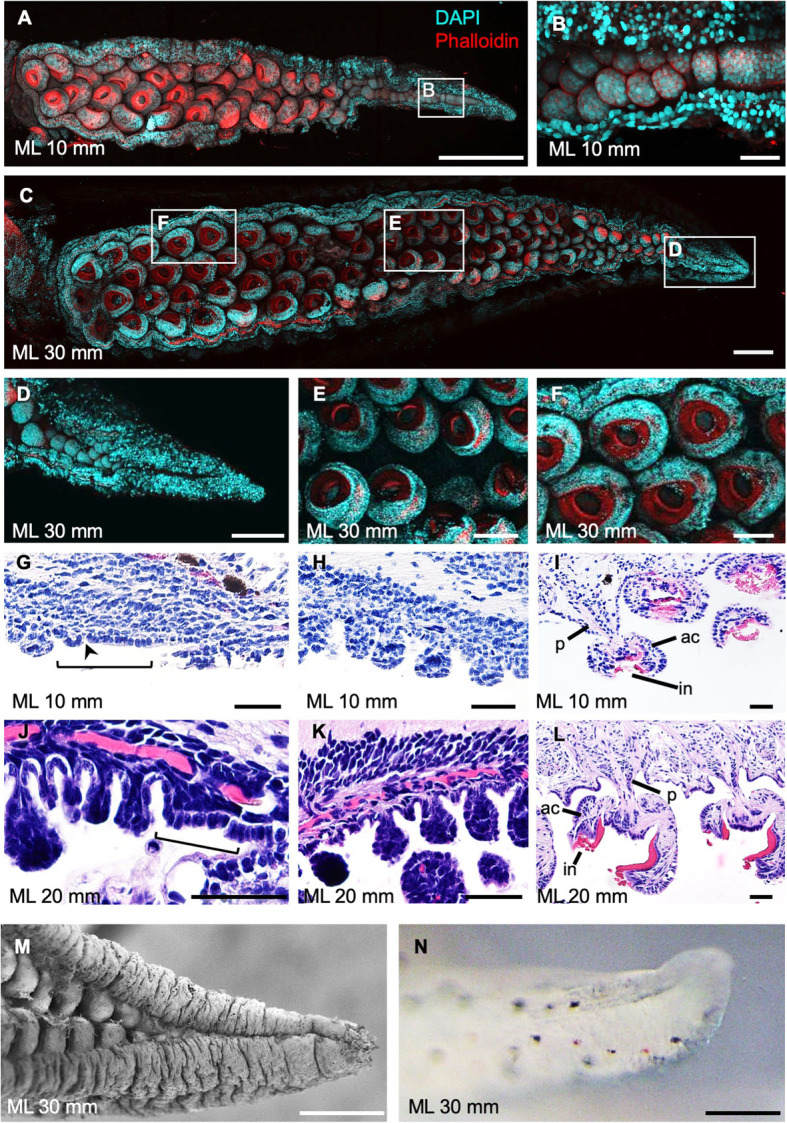
The postembryonic process of sucker formation of the second arm in *S. lycidas*. Arms are oriented with distal to the right. **a**-**f** Confocal stacks of arms from oral view. **a** Second arm of an individual with ML (mantle length) 10 mm (n = 3). **b** Higher magnification of the white boxed region in A. **c** an individual of ML 30 mm (n = 3). **d**-**f** Higher magnification of the white boxed regions in C. **g**-**l** Histological sections in sagittal planes of an individual with ML 10 mm (**g**-**i**) and ML 20 mm (**j**-**l**), stained with hematoxylin and eosin; distal (**g**, **j**), middle (**h**, **k**) and proximal (**i**, **l**) parts. (**m**, **n**) The epithelium covering the arm tip in an individual with ML 30 mm; SEM observation (**m**) and live specimen (**n**). Scale bars indicate 500 μm (**a**, **c**), 200 μm (**d**-**f**, **m**, **n**), and 50 μm (**b**, **g**-**l**). Abbreviations: ac, acetabulum; in, infundibulum; p, peduncle

The external morphologies of arms and suckers in juveniles of *S. esculenta* and *S. lycidas*, observed by CLSM, showed that small dome-shaped sucker buds were seen only in the distal part of the arm, and larger suckers in the proximal arm part seemed to have functional structures like those seen in adult cuttlefishes (Fig. 5a-f, Additional file 4: Fig. S4). Histological observations showed that the sucker size was larger in the proximal part than that in the distal part. Most suckers, except for sucker buds in the distal tip, had distinct adult sucker structures, such as infundibulum, acetabulum and peduncle (Fig. 5g-l). As in embryos, at the most distal sucker field ridge a single epithelial layer was observed on the oral side of an arm tip, in which no clear tissue structures were seen (Fig. 5g, h, j, k).

Furthermore, SEM observations of the external morphology revealed that the epithelial tissues were expanded from the side of the arm’s tip, covering the undifferentiated area at the distal end of the tip. This sheath-like structure was observed throughout postembryonic development (Fig. 5m, Additional file 5: Fig. S5). The sucker field ridge and dome-shaped sucker buds were completely covered by the epithelial sheath. The epithelial sheath cover was also confirmed by observations on live specimens, to exclude the possibility that the epithelia had shrunk due to fixation for SEM observations (Fig. 5n, Additional file 5: Fig. S5).

## Discussion

**Fig. 6:**
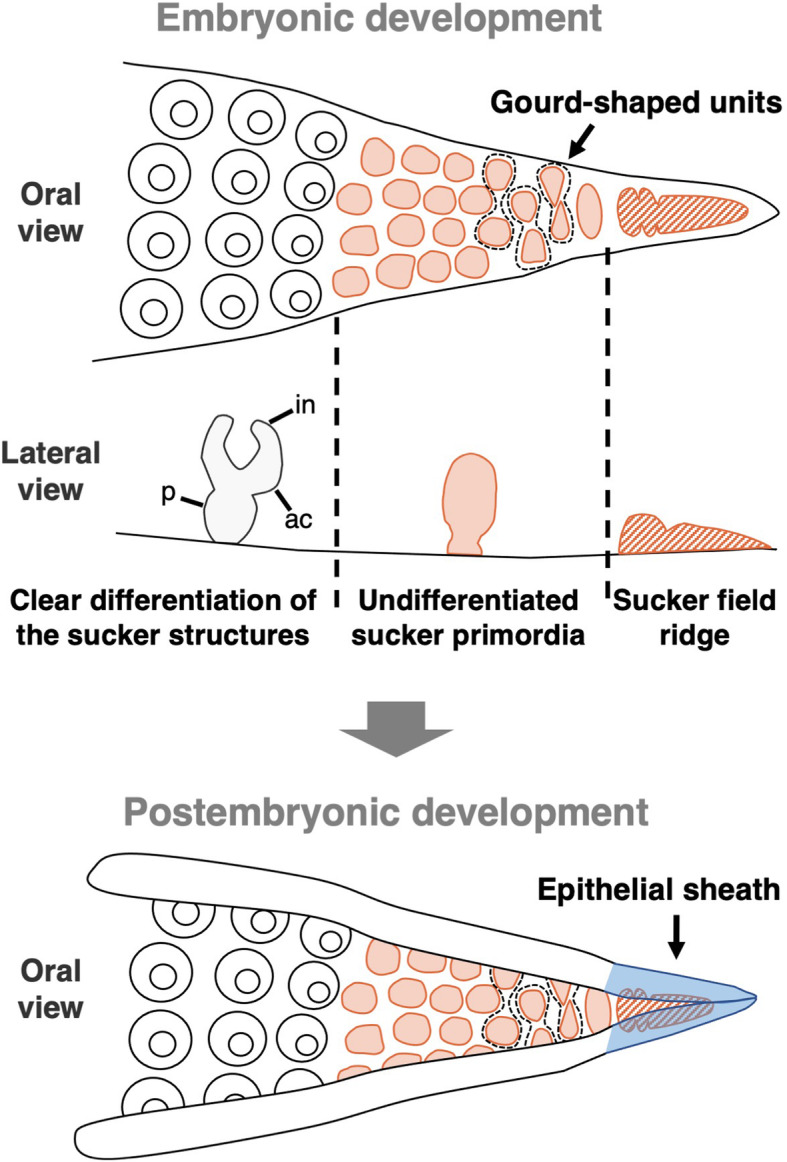
A schematic diagram of sucker formation during embryonic and postembryonic development. The second arm in *Sepia* is focused on. Abbreviations: ac, acetabulum; in, infundibulum; p, peduncle

This study revealed the morphogenetic process of suckers on the second arm during embryonic and postembryonic development that are shared between 2 cuttlefish species, *S. esculenta* and *S. lycidas* (Fig. 6). As seen in other Coleoidea (octopuses, squids and cuttlefishes), the focal cuttlefishes possess numerous small suckers that are located at the distal tips of arms. This suggests that the suckers are newly formed at the distal tips. Firstly, the pattern of the increase of the number of suckers in a second arm was investigated in this study, and the results indicated that the suckers are newly added during embryonic and postembryonic development (none at St. 25, about 50 suckers/arm at hatching, about 200 suckers/arm in mature cuttlefish; Fig. 2).

In adult cuttlefishes, there were almost no structural differences of suckers between the proximal and distal part of arm. However, the sucker sizes were different between the two parts; suckers in proximal part were larger than those in distal part, suggesting that the sucker function might be different between the two. It was reported that the adhesion strength varied depending on sucker sizes [32], indicating that cephalopods may properly use suckers in different sizes at different positions [33].

We found that during embryogenesis, at the oral side of the most distal tip of the arm, a long narrow ridge (sucker field ridge), followed by dome-shaped sucker buds without clear inner structures, was observed, while at the more proximal part, primordial suckers that possessed differentiated sucker structures were observed, as also seen in adult cuttlefishes. This strongly suggests that undifferentiated suckers are newly formed in the distal part of an arm and they develop to have functional sucker structures as they become located in a relatively more proximal part, in association with the arm elongation. The sucker field ridge at the arm tip was constricted at its proximal side, adjacent to a mass of dome-shaped sucker buds, suggesting that new sucker buds were produced and pinched off from the sucker field ridge. These small buds lined up in the distal part of the arms were also reported in *S. officinalis* [21]. Therefore, it is suggested that, at least in the genus *Sepia*, the sucker formation pattern in which suckers are formed at the most distal arm tip during the arm elongation, is shared among species.

Adjacent to the proximal end of the sucker field ridge, there were gourd-shaped bulges that were visualized by actin localization (Figs. 3f, g, 4e, f). This suggested that the gourd-shaped bulge is separated from the sucker field ridge, and that it divides into two dome-shaped buds that become two primordial suckers. Pairs of primordial suckers derived from two gourd-shaped bulges were aligned in a staggered array to form 4-sucker rows in the proximal part. This arrangement of sucker row formation seems consistent with the fact that, in Coleoidea, the number of suckers per row in adults is 2^n^ in many species [10].

Although the general process of sucker formation was similar between *S. esculenta* and *S. lycidas*, some differences were found especially in the timing and speed of sucker development as seen in the increase of sucker number (Fig. 2b) and the process of sucker differentiation (Figs. 3a-e, 4a-c). These indicate that there is a heterochronic shift between these two species.

The process of sucker formation during postembryonic development showed a similar pattern to that observed during embryogenesis (Fig. 5a-l, Additional file 4: Fig. S4). In the postembryonic development, however, primordial suckers on the arm tip were covered by epithelial sheaths that extended from both sides of the arm tip (Fig. 5m, n, Additional file 5: Fig. S5), probably for protecting the undifferentiated, vulnerable primordial suckers, since cuttlefish juveniles after hatching are exposed to the external environment. This epithelial expansion was not seen during embryonic development, and thus the expansion might start in response to some stimuli from external environment.

In this study, the second arms of cuttlefishes were mainly studied. However, since the number of suckers and/or the arrangement can be largely different among arms and tentacles [15, 18], the differences of sucker development among these body parts should also be compared to better understand the fundamental process of sucker formation in future studies. Also, as this study just revealed the basic developmental patterns of suckers, in future studies, it will be necessary to investigate the detailed differentiation processes of suckers, for example, by applying molecular markers or gene expression analyses.

The sucker morphologies and the number of sucker rows are diversified among cephalopod species [15, 34]. However, the ancestral state of suckers cannot be inferred, since all the extant Coleoidea species (octopuses, squids and cuttlefishes) possess well-developed suckers on their arms [35], while the extant Nautilidea species (nautliuses) completely lack suckers [10], and there are no intermediate cephalopod species. A few studies have been performed on the sucker development in squids and cuttlefishes. In *E. scolopes* (Sepiolida), for example, it is reported that small undifferentiated suckers are observed on the oral side of the arm tip, indicating that new suckers are formed at the distal arm tip [20]. Together with our data, it is suggested that the patterns of sucker development are shared with Sepiida.

The sucker formation during embryogenesis in octopus (Octopodiformes) has also been studied, and in octopus, remarkably fewer suckers are formed before hatching than in cuttlefishes: 3 suckers in *Argonauta argo* and 8 suckers in *Eledone cirrosa* develop in a row [36, 37]. Thus, the patterns of sucker formation in octopus seem to be different from those in squids and cuttlefishes. Furthermore, although the onset of sucker formation at the arm tip seemed to be shared between octopuses and cuttlefishes, the detailed differentiation process of sucker structures such as basal constriction to peduncle formation and actin localization around the rim of infundibulum to sucker ring teeth formation were largely different [38].

In addition, it could be assumed that the diversification of sucker morphologies might contribute to the adaptive radiation of cephalopods, in which benthic or pelagic lifestyles and diverse habitats have been acquired [39]. In that sense, nautliuses (Nautilidea) attract special attention since they have many arms without any suckers. In Nautilidea, the number of species is small and the habitat is restricted to warm-water areas [12], supporting the idea that sucker acquisition contributes to adaptive radiation. Thus, comparisons among species across the cephalopod lineage will be required to understand the evolutionary patterns of sucker formation and functions in Cephalopoda.

## Conclusions

In this study, to elucidate the pattern of sucker formation, morphological and histological observations were carried out during embryonic and postembryonic development in *S. esculenta* and *S. lycidas*, focusing on the distal arm tips, at which suckers were shown to be newly formed. The observations showed that suckers were newly formed in the distal arm tips and functional sucker structures were differentiated as they become located in a relatively proximal region due to the arm elongation. Although in this study the sucker formation pattern was revealed in *Sepia*, the sucker morphologies are diversified among cephalopod species and the ancestral state of suckers cannot be inferred, since there are no cephalopod species with intermediate sucker morphologies. Therefore, comparisons among species across the cephalopod lineage will be necessary to reveal the evolutionary patterns of sucker formation in Cephalopoda.

## Methods

### Animals

Sexually mature adults of *S. esculenta* and *S. lycidas* were captured in Sagami bay by nets set off the coast of Okusu fishing port in Yokosuka city, Japan, in April and May in 2019 and 2020. Collected cuttlefishes were maintained in aquaria (1240 × 760 × 550 mm, 3 individuals per aquarium) with circulating sea water at 14–16 °C for about a month (Additional file 6: Fig. S6A) and fed frozen Antarctic krill. These aquaria were placed in a room under constant light condition even during the night, to avoid sudden light on/off due to human activities, which may cause stress to cuttlefishes. Cylindrical polyethylene nets were used as spawning beds, on which fertilized eggs were laid (Additional file 6: Fig. S6A). Fertilized eggs were collected and transferred into another aquarium, in which the water temperature was gradually raised to 21 °C because higher temperature is known to promote cuttlefish embryogenesis [40].

After hatching, juveniles were maintained in a plastic container (715 × 410 × 200 mm) with an aeration apparatus and running sea water under constant light condition (Additional file 6: Fig. S6B). The sea water temperature rose up to 28 °C. These juveniles were reared for 3 months at longest. Juvenile cuttlefishes were fed small mysids (Mysidae gen. sp.), shrimps (e.g., *Heptacarpus futilirostris, Palaemon serrifer and Lysmata vittata*) and crabs (e.g., *Gaetice depressus*), captured from coastal areas around Misaki Marine Biological Station. Embryos and juveniles were observed using a stereomicroscope (SZX16; Olympus, Tokyo, Japan), equipped with a digital camera (DP50; Olympus, Tokyo, Japan). The number of suckers after hatching were counted based on the captured images.

### Fluorescent staining of nuclei and cytoskeletons

Fixation and fluorescent staining were performed according to previous studies (e.g., [41]). Briefly, embryos at each developmental stage and post-hatch juveniles with mantle length of 10–40 mm were fixed in 4% paraformaldehyde (PFA) in filtered sea water (FSW) for 1 to 3 h after they were anesthetized with 7% MgCl_2_. The fixed samples were then preserved in 0.3% Triton-X 100 in 1× phosphate-buffered saline (PBT) at 4 °C until use. For post-hatch juveniles, the arm epithelium that covers suckers was removed under a stereoscopic microscope before the preparation, since it would be an obstacle for CLSM observations. The fixed samples were washed in PBT for 15 min at least three times before staining. The nuclei (DNA) and cytoskeletons (F-actin) were respectively stained with 4′,6-diamidino-2-phenylindole (DAPI) (2 μg/ mL; Sigma, St Louis, MO, USA) and rhodamine-phalloidin (1:40; Invitrogen, Paisley, UK), for 1 h at room temperature, and stained samples were then washed for 15 min in PBT at least three times. Stained samples (*n* = 3–5) were observed under a CLSM (FV3000; Olympus, Tokyo, Japan). Data on the sucker number increase during embryogenesis were obtained, based on the fluorescent images.

### Histological analysis

To histologically observe the inner structures of suckers, paraffin sections were made according to the method described in previous studies (e.g., [42]). Embryos of *S. esculenta* and *S. lycidas* in each developmental stage and post-hatch juveniles of *S. lycidas* with mantle length of 10–40 mm were fixed in Bouin’s solution (saturated aqueous picric acid solution/ formalin/ acetic acid = 15:5:1) or 4% PFA in FSW for longer than 8 h after they were anesthetized with 7% MgCl_2_. Then, fixed samples were preserved in 70% EtOH until use. Samples were dehydrated in increasing concentrations of ethanol, then transferred into xylene and finally embedded into paraffin. Serial sections (5–7 μm thick) of sagittal planes were prepared with a microtome (Spencer Lens Co., Buffalo, USA) and stained with hematoxylin and eosin. Tissues on slides were observed using an optical microscope (BX51; Olympus, Tokyo, Japan) and photographs were taken using a digital camera (DP74; Olympus, Tokyo, Japan) attached to the microscope.

### Observations of external morphology

Scanning electron microscopy was carried out to investigate the external morphology of the arm tip. Juveniles with mantle length of 20 mm, 30 mm or 40 mm were fixed in Bouin’s solution for more than 8 h after they were anesthetized with 7% MgCl_2_, and then preserved in 70% EtOH until use. Then, the arms of fixed specimens were dehydrated in increasing concentrations of ethanol and transferred into hexamethyldisilazane for 1 h, and then into *t*-butanol. After that, the fixed samples were freeze-dried using a Freeze Dryer ES-2030 (Hitachi Global, Tokyo, Japan), and coated with silver ions with an Ion Sputter E-1010 (Hitachi Global, Tokyo, Japan). Coated samples were observed with a JSM-5510LV scanning electron microscope (JEOL Ltd., Tokyo, Japan). Additionally, in order to exclude the possibility of shrinkage by the fixation process, the arms of live specimens were also observed with a stereomicroscope (SZX16; Olympus, Tokyo, Japan). Photographs were taken with a digital camera (DP50; Olympus, Tokyo, Japan) attached to the stereomicroscope.

## Supplementary information

**Additional file 1: Figure S1.** A schematic image of developmental stages during embryogenesis, based on Yamamoto (1982). (JPEG 162 kb)

**Additional file 2: Figure S2.** Optical sections (horizontal) at the base of sucker buds in *S. esculenta* and *S. lycidas*. Arms are oriented with distal to the right. (A-D) *S. esculenta* at St. 34–36 (A, B; *n* = 5) and at St. 38–39 (C, D; n = 5). (E-H) *S. lycidas* at St. 34–36 (E, F; *n* = 4) and St. 38–39 (G, H; n = 5). White frames indicate the gourd-shaped actin localization. Scale bars indicate 50 μm. (JPEG 459 kb)

**Additional file 3: Figure S3.** Optical sections of the second arm in *S. esculenta* (St. 34–36) in the horizontal planes. Sections are obtained from the planes in which each primordial section area is largest. Scale bars indicate 50 μm; *n* = 5. (JPEG 258 kb)

**Additional file 4: Figure S4.** The postembryonic process of sucker formation of the second arm in *S. esculenta*. Arms are oriented with distal to the right. (A-F) Confocal stacks of arms from oral view. (A) Second arm of an individual with ML (mantle length) 10 mm (n = 4). (B) Higher magnification of the white boxed region in A. (C) An individual of ML 20 mm (n = 4). (D-F) Higher magnification of the white boxed regions in C. Scale bars indicate 200 μm (A, C) and 50 μm (B, D-F). (JPEG 532 kb)

**Additional file 5: Figure S5.** Epithelia covering the arm tip in *S. lycidas*. Arms are oriented with distal to the right. (A, C) SEM images. (B, D) Live specimens. Individuals with mantle length of 20 mm (A, B) and 40 mm (C, D) were used. Scale bars indicate 100 μm. (JPEG 406 kb)

**Additional file 6: Figure S6.** The rearing system of cuttlefishes. A: adults. Arrow indicates a spawning bed. B: juveniles after hatching. (JPEG 207 kb)

## Data Availability

The datasets generated during and/or analyzed during the current study are available from the corresponding author on reasonable request.

## Abbreviations

ac: Acetabulum
in: Infundibulum
p: Peduncle
srt: Sucker ring teeth
n: Nerve
ML: Mantle length
