# Supplementary figures and images for: Pattern of sucker development in cuttlefishes
Source: Front Zool. 2020 Aug 24;17:24. doi: 10.1186/s12983-020-00371-z (PMC7444262; doi:10.1186/s12983-020-00371-z)

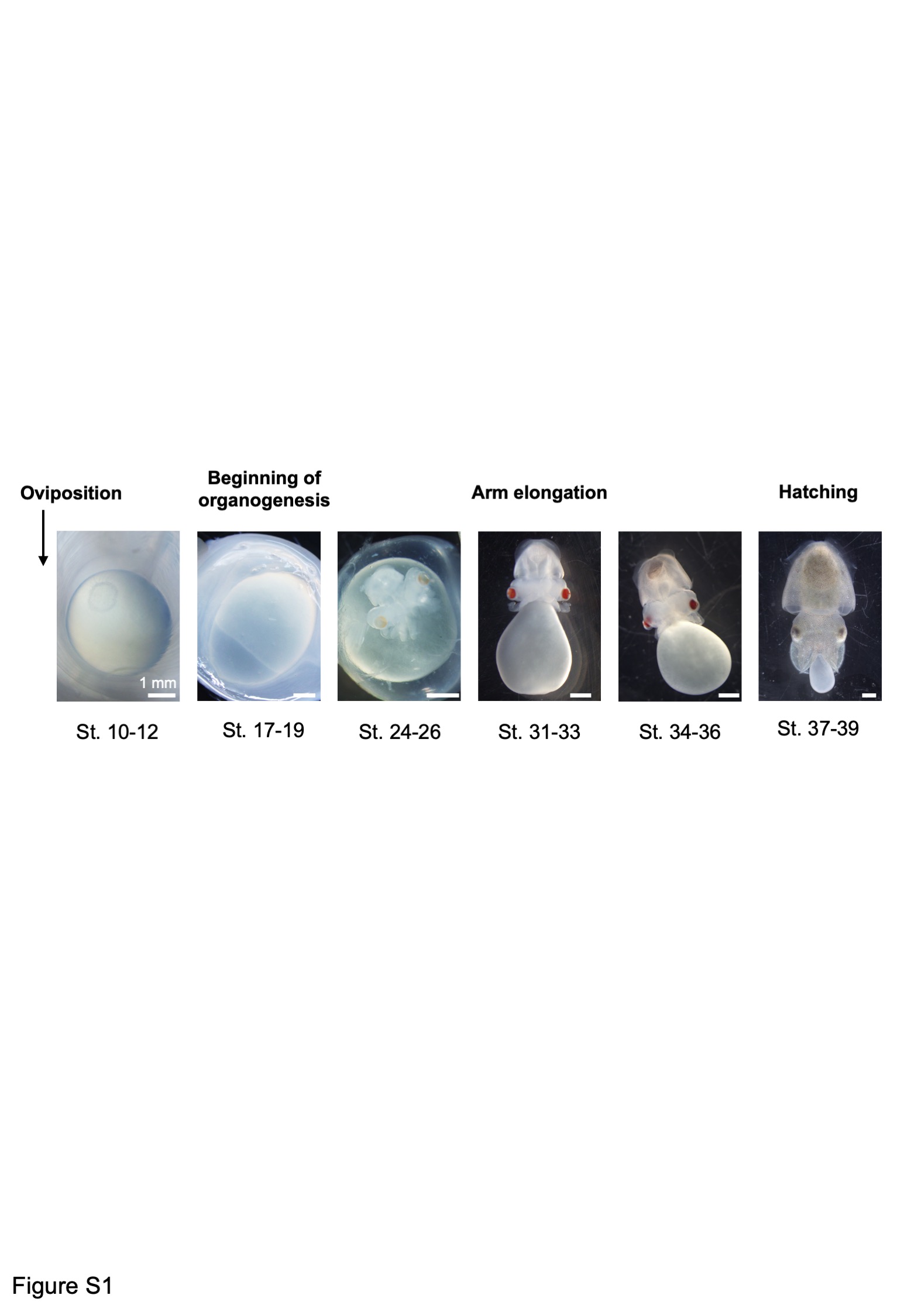

Supplement: Supplementary file 1 — Additional file 1: Figure S1. A schematic image of developmental stages during embryogenesis, based on Yamamoto (1982). (JPEG 162 kb) [file 12983_2020_371_MOESM1_ESM.jpeg]

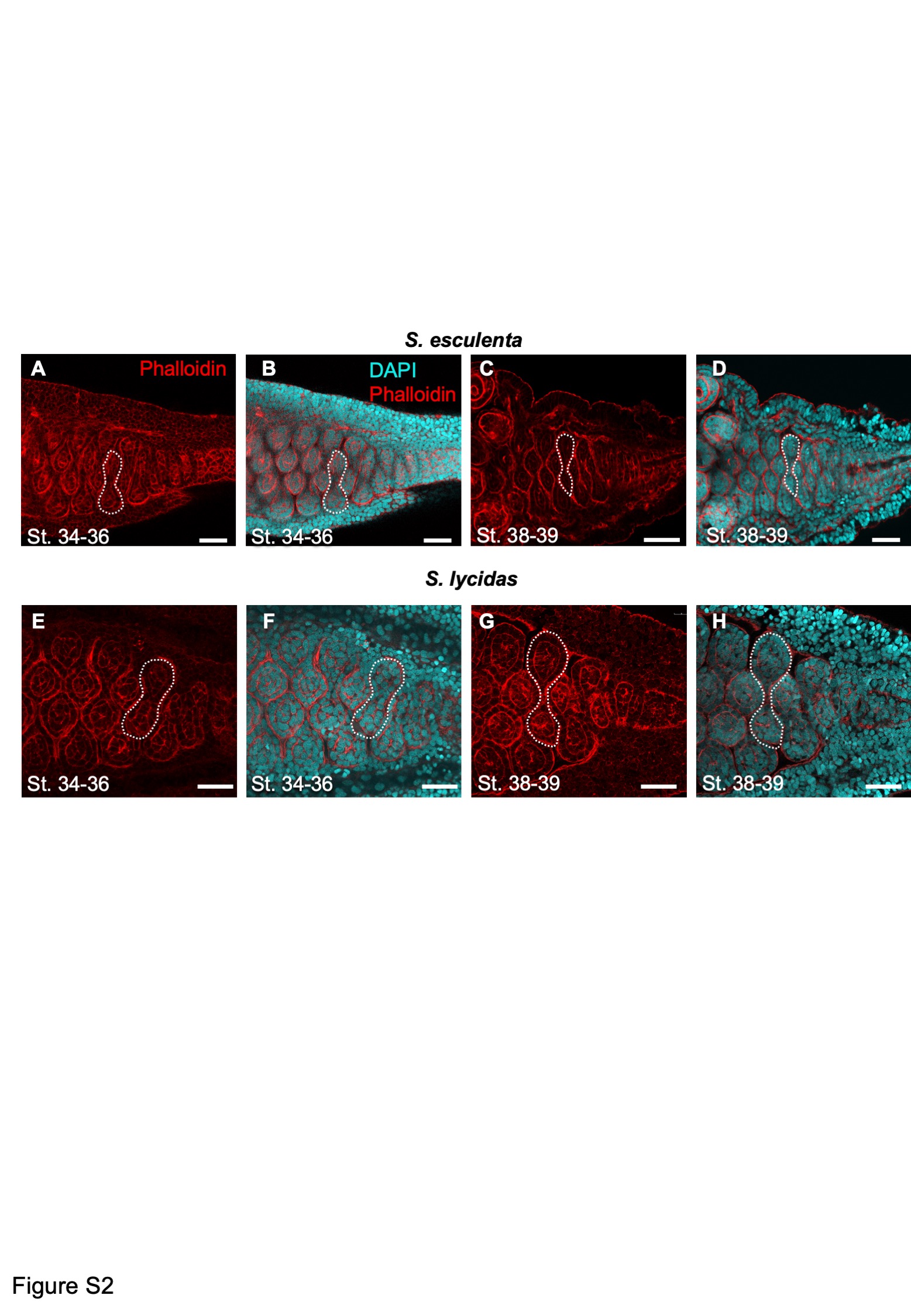

Supplement: Supplementary file 2 — Additional file 2: Figure S2. Optical sections (horizontal) at the base of sucker buds in S. esculenta and S. lycidas. Arms are oriented with distal to the right. (A-D) S. esculenta at St. 34–36 (A, B; n = 5) and at St. 38–39 (C, D; n = 5). (E-H) S. lycidas at St. 34–36 (E, F; n = 4) and St. 38–39 (G, H; n = 5). White frames indicate the gourd-shaped actin localization. Scale bars indicate 50 μm. (JPEG 459 kb) [file 12983_2020_371_MOESM2_ESM.jpeg]

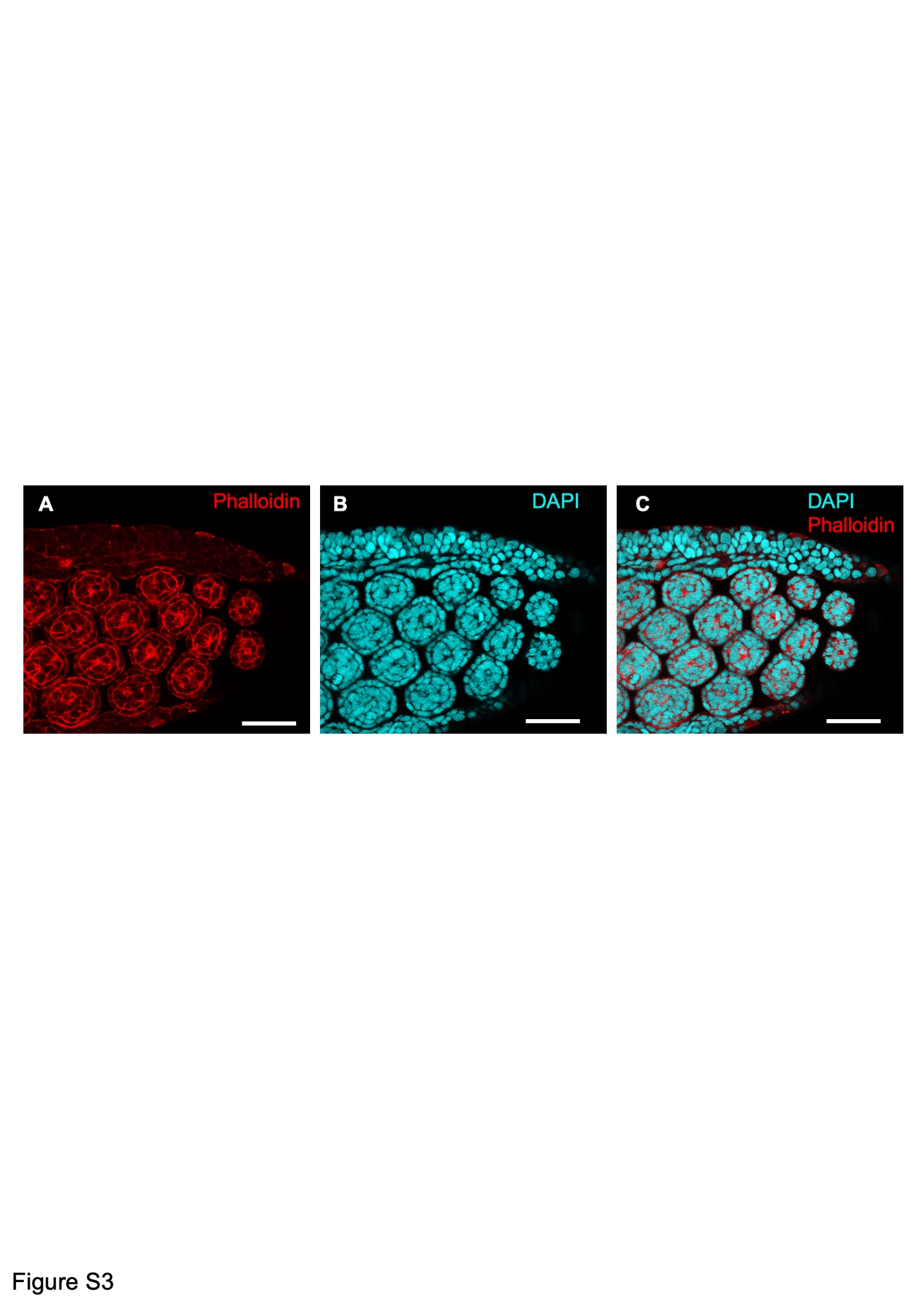

Supplement: Supplementary file 3 — Additional file 3: Figure S3. Optical sections of the second arm in S. esculenta (St. 34–36) in the horizontal planes. Sections are obtained from the planes in which each primordial section area is largest. Scale bars indicate 50 μm; n = 5. (JPEG 258 kb) [file 12983_2020_371_MOESM3_ESM.jpeg]

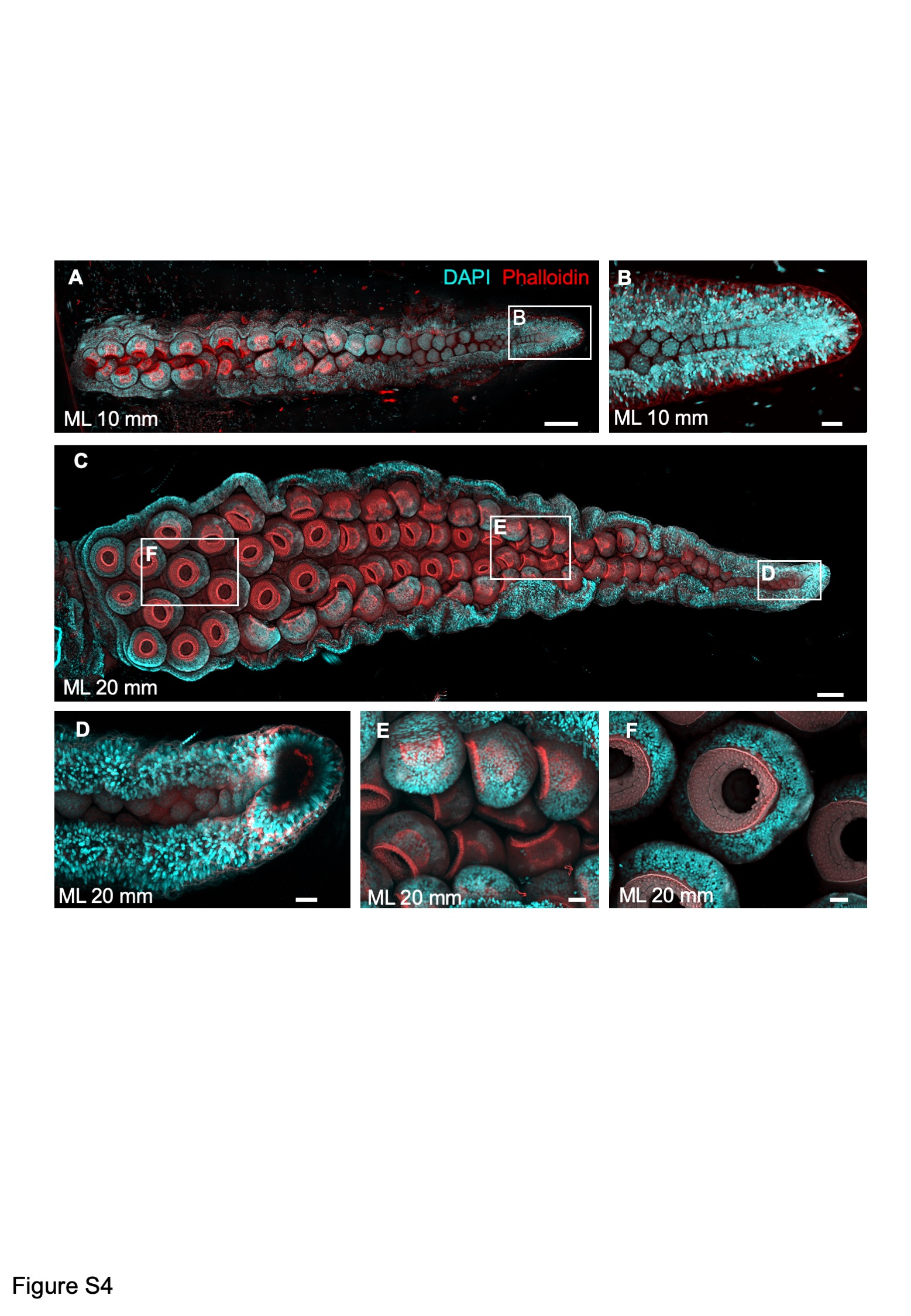

Supplement: Supplementary file 4 — Additional file 4: Figure S4. The postembryonic process of sucker formation of the second arm in S. esculenta. Arms are oriented with distal to the right. (A-F) Confocal stacks of arms from oral view. (A) Second arm of an individual with ML (mantle length) 10 mm (n = 4). (B) Higher magnification of the white boxed region in A. (C) An individual of ML 20 mm (n = 4). (D-F) Higher magnification of the white boxed regions in C. Scale bars indicate 200 μm (A, C) and 50 μm (B, D-F). (JPEG 532 kb) [file 12983_2020_371_MOESM4_ESM.jpeg]

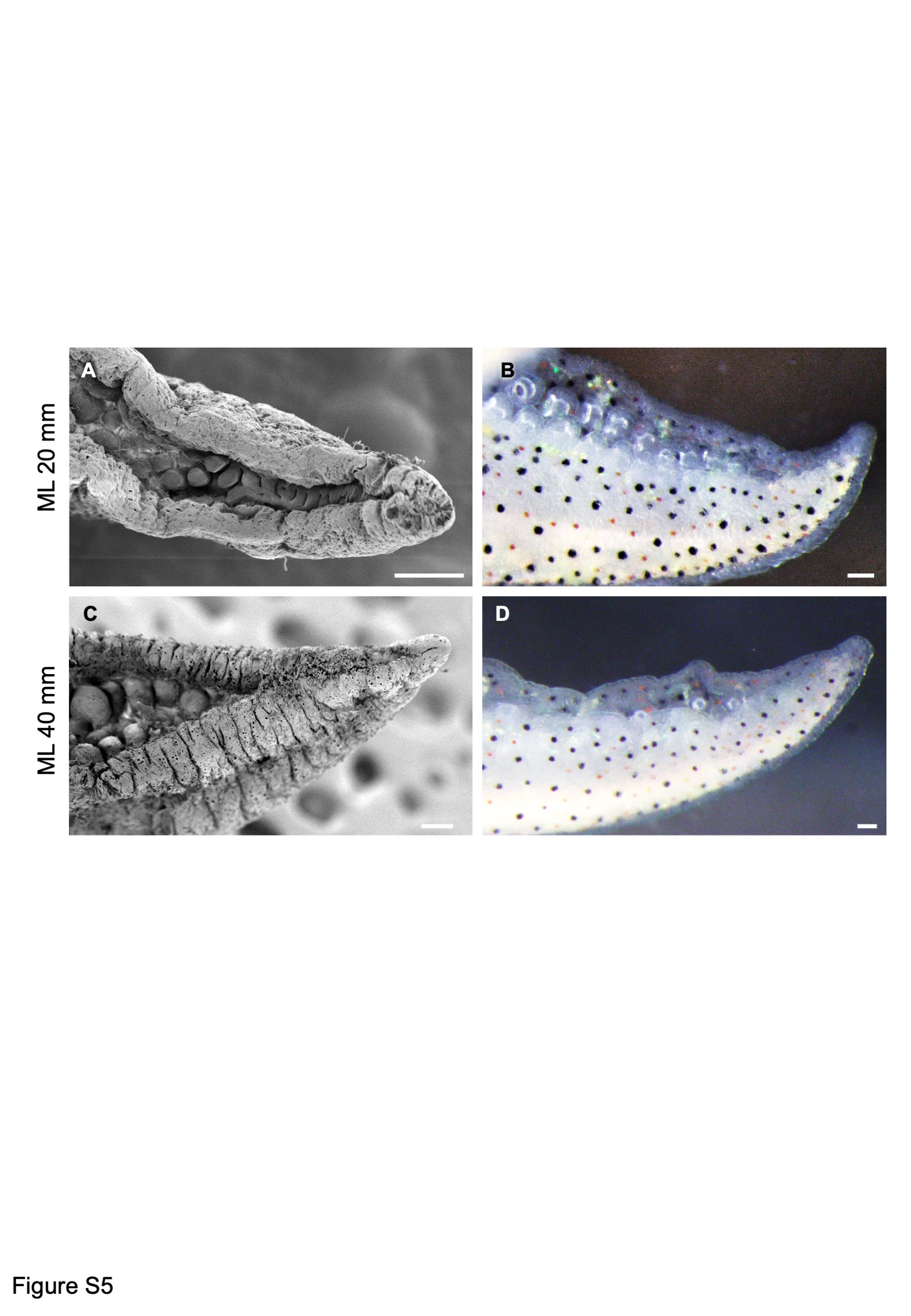

Supplement: Supplementary file 5 — Additional file 5: Figure S5. Epithelia covering the arm tip in S. lycidas. Arms are oriented with distal to the right. (A, C) SEM images. (B, D) Live specimens. Individuals with mantle length of 20 mm (A, B) and 40 mm (C, D) were used. Scale bars indicate 100 μm. (JPEG 406 kb) [file 12983_2020_371_MOESM5_ESM.jpeg]

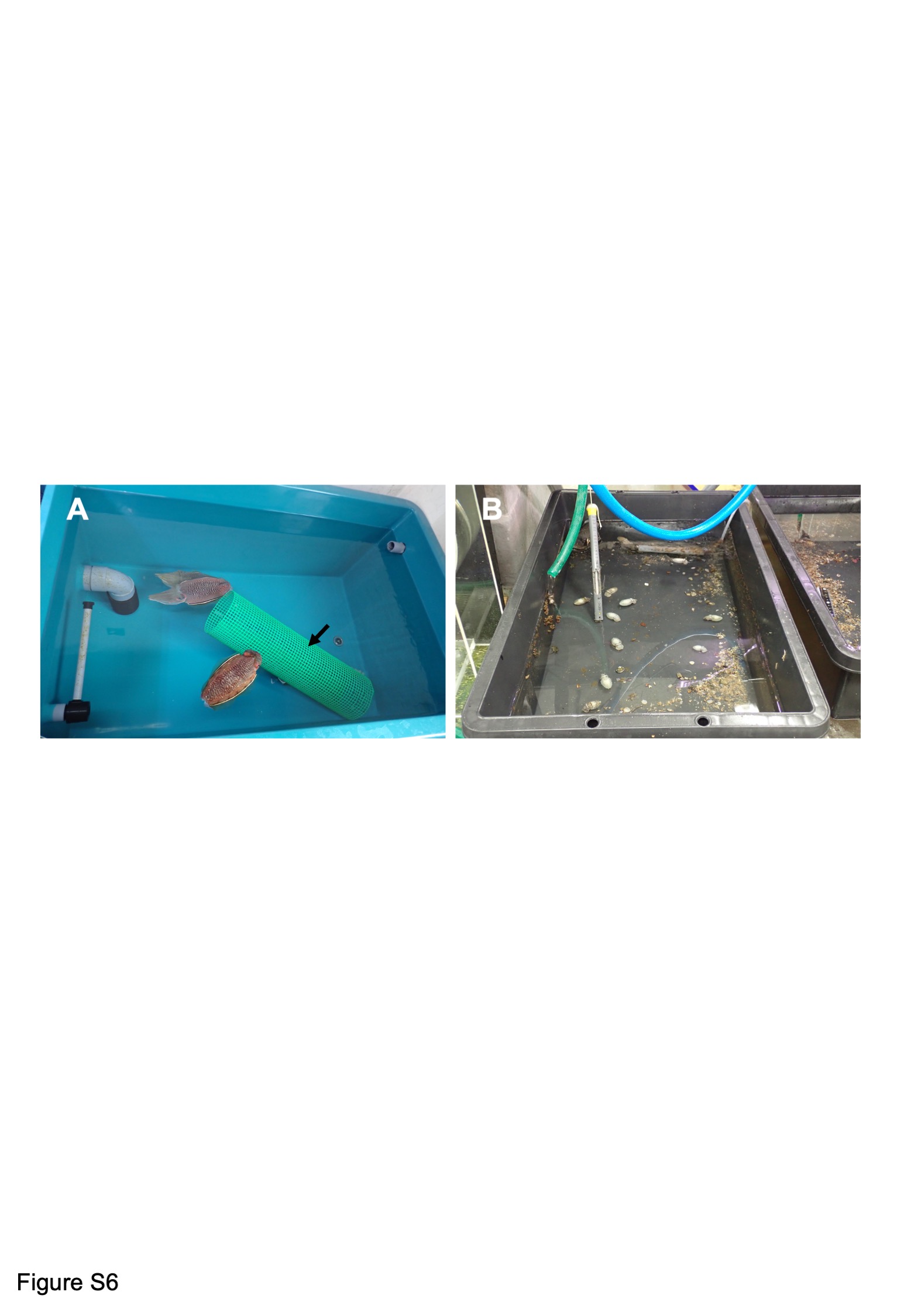

Supplement: Supplementary file 6 — Additional file 6: Figure S6. The rearing system of cuttlefishes. A: adults. Arrow indicates a spawning bed. B: juveniles after hatching. (JPEG 207 kb) [file 12983_2020_371_MOESM6_ESM.jpeg]
